# Two-frequency chimera state in a ring of nonlocally coupled Brusselators

**DOI:** 10.1371/journal.pone.0187067

**Published:** 2017-10-27

**Authors:** Qionglin Dai, Danna Liu, Hongyan Cheng, Haihong Li, Junzhong Yang

**Affiliations:** School of Science, Beijing University of Posts and Telecommunications, Beijing, China; Hong Kong Baptist University, HONG KONG

## Abstract

Chimera states, which consist of coexisting domains of spatially coherent and incoherent dynamics, have been intensively investigated in the past decade. In this work, we report a special chimera state, 2-frequency chimera state, in one-dimensional ring of nonlocally coupled Brusselators. In a 2-frequency chimera state, there exist two types of coherent domains and oscillators in different types of coherent domains have different mean phase velocities. We present the stability diagram of 2-frequency chimera state and study the transition between the 2-frequency chimera state and an ordinary 2-cluster chimera state.

## Introduction

Chimera state refers to a type of fascinating hybrid dynamical states in which identically coupled units spontaneously develop into coexisting synchronous and asynchronous parts. Since its discovery in nonlocally coupled phase oscillators in 2002 [1], chimera state has become a very active research field [2, 3]. It has been extensively observed that chimera states can occur in globally coupled [4, 5] and locally coupled oscillators [6], periodic and chaotic maps [7], Stuart-Landau models [8, 9], Van der Pol oscillators [10], FitzHugh-Nagumo (FHN) oscillators [11], Hindmarsh-Rose models [12], Hodgkin-Huxley models [13] and Delayed-Feedback Systems [14]. Chimera states on random networks and on multiplex networks have been investigated [15–17]. Recently, chimera states were realized experimentally in chemical [18, 19], optical [20, 21], electronical [14], mechanical and electrochemical systems [22–25].

Different types of chimera states such as breathing chimeras [2], multi-cluster chimeras [26–28], and spiral chimeras [29, 30] have been discovered and investigated in details. However, in these chimera states, coherent oscillators always have the same mean phase velocity. In this work, we will report a new type of chimera state in which coherent oscillators may have different mean phase velocities.

## Materials and methods

We consider a one-dimensional ring of *N* nonlocally coupled Brusselator [31] in which the individual unit is coupled to *R* neighbors on each side with coupling strength *ϵ*:

$$\begin{array}{ccc} \dot{X_{k}} & = & A-\left(B+1\right)X_{k}+X_{k}^{2}Y_{k}\\ & & +\frac{\epsilon }{2R}\;\sum_{j=k-R}^{k+R}\;D_{uu}\left(X_{j}-X_{k}\right)+D_{uv}\left(Y_{j}-Y_{k}\right),\\ \dot{Y_{k}} & = & BX_{k}-X_{k}^{2}Y_{k}\\ & & +\frac{\epsilon }{2R}\;\sum_{j=k-R}^{k+R}\;D_{vu}\left(X_{j}-X_{k}\right)+D_{vv}\left(Y_{j}-Y_{k}\right) \end{array}$$ (1)

The subscript *k* refers to the unit index, which has to be taken module *N* (or period boundary condition). Following Ref. [11], the coupling matrix is modelled as:

$$\begin{array}{c} D=(\begin{array}{cc} D_{uu} & D_{uv}\\ D_{vu} & D_{vv} \end{array})=(\begin{array}{cc} \cos\phi & \sin\phi \\ -\sin\phi & \cos\phi \end{array}). \end{array}$$ (2)

Brusellator is a theoretical model for a type of autocatalytic reaction. Isolated Brusselator allows for an equilibrium at *X* = *A* and *Y* = *B*/*A*. When *B* > 1 + *A*^2^, the equilibrium becomes unstable and leads to a limit cycle.

To require that the Brusselator units work in the oscillatory regime, we set *A* = 1, *B* = 2.1. It is convenient to consider the ratio *r* = *R*/*N*, the coupling radius, which ranges from 1/*N* (nearest-neighbor coupling) to 0.5 (global coupling). In addition, we let *ϕ* = *π*/2 + *θ*. Throughout the paper, we numerically simulate Eq (1) by using the fourth-order Runge-Kutta method with a time step *δt* = 0.01. The total number of the Brusselator units is set to *N* = 1000.

## Results and discussion

**Fig 1:**
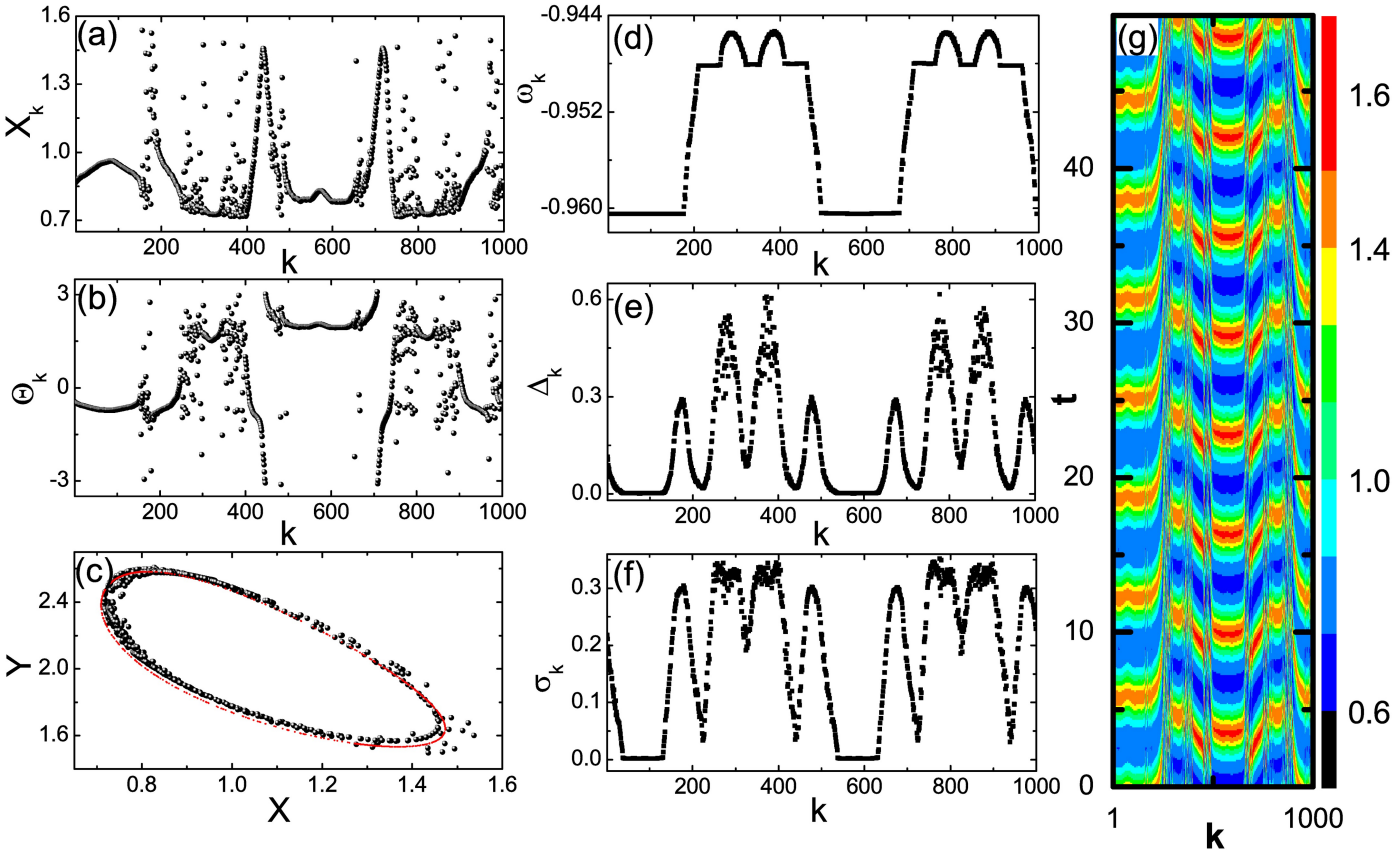
Two-frequency chimera state. (a) Snapshot of the variable *X*_*k*_. (b) Snapshot of the phase Θ_*k*_. (c) Snapshot of oscillators in the (*X*, *Y*) plane (in black). Red curve denotes the orbit of isolated Brusselator. (d) The profile of the mean phase velocity *ω*_*k*_. (e) The profile of the difference between adjacent oscillators Δ_*k*_. (f) The profile of the variance *σ*_*k*_. (g) The spatiotemporal plot of the variable *X*_*k*_ after transient time. *A* = 1, *B* = 2.1, *r* = 0.35, *θ* = −0.1, and *ϵ* = 0.02.

We report a peculiar chimera state at *r* = 0.35, *θ* = −0.1, and *ϵ* = 0.02 in Fig 1. The snapshot of the variable *X*_*k*_ in (a) and the snapshot of the phase of oscillator Θ_*k*_, defined as $e^{i\Theta_{k}}=\left(\dot{X_{k}}+i\dot{Y_{k}}\right)/\left|\dot{X_{k}}+i\dot{Y_{k}}\right|$, in (b) show the coexistence of spatially coherent domains, in which oscillators distribute their variables in space in a continuous way, and incoherent domains, in which the variables of oscillators are scattered. There exist two large and several small coherent domains. Oscillators in the same large coherent domain are nearly in phase while those in different large coherent domains have a phase difference between them at around *π*. In contrast, coherent oscillators in small coherent domains may disperse their variables over a large range such as the phase in the range of 2*π*. The snapshot of the oscillators in the (*X*, *Y*) plane in Fig 1(c) shows that oscillators do not fall onto the orbit of isolated oscillators.

Coherent and incoherent domains can be identified more clearly by the mean phase velocity of oscillators which is defined as *ω*_*k*_ = lim_*t*−*t*′ → ∞_[Θ_*k*_(*t*) − Θ_*k*_(*t*′)]/(*t* − *t*′) with *t*′ the transient time. Oscillators in a same coherent domain share the same mean phase velocity while those in a same incoherent cluster have different mean phase velocities. As shown by the profile of *ω*_*k*_ in Fig 1(d), there exists two large coherent domains and six small coherent domains. In an ordinary view on chimera state containing multi-coherent-cluster, all coherent oscillators share the same mean phase velocity. However, Fig 1(d) shows an extraordinary feature: oscillators in the two large coherent domains share a same mean phase velocity Ω_1_ while those in the other six small coherent domains share another mean phase velocity Ω_2_. Ω_1_ ≠ Ω_2_ suggests that there are no fixed phase difference between oscillators in the large and the small coherent domains. From now on, we call the chimera state as 2-frequency chimera state. The profile of the mean phase velocity shows that the six small coherent domains are partitioned evenly into two groups and spatially separated by the large coherent domains. Regardless of the antiphase between the two large coherent domains, the 2-frequency chimera state is symmetric in space under the transformation, *k* → 2*k*_0_ − *k* with *k*_0_ the location of the center of the large coherent domain or the location of the center of the middle one among the three adjacent small coherent domains (For convenience, we call the middle one in the adjacent three small coherent domains as M-domain and others as S-domains). Thereby, the coherent domains are classified as the large domain, the M-domain, and the S-domain. Within the same type of coherent domain, different domains have the same domain size.

To further characterize the 2-frequency chimera state, we consider two other measures. One is the difference between adjacent oscillators, defined as $\Delta_{k}={\langle \Delta_{k}\left(t\right)\rangle }_{t}={\langle \sqrt{{\left(X_{k}-X_{k+1}\right)}^{2}+{\left(Y_{k}-Y_{k+1}\right)}^{2}}\rangle }_{t}$ with 〈⋅〉_*t*_ the time average and the other is the variance *σ*_*k*_ of Δ_*k*_(*t*). The profile of Δ_*k*_ in Fig 1(e) shows that Δ_*k*_ reaches its minima in coherent domains. Δ_*k*_ is nearly zero in the two large coherent domains, which confirms that coherent oscillators in the same large domain are almost in phase. On the other hand, Δ_*k*_ stays at nonzero values in both M-domains and S-domains, which is in agreement with the observation that oscillators in small coherent domains are off phase as shown in Fig 1(a) and 1(b). Actually, Δ_*k*_ fluctuates with the locations of oscillators in the incoherent domains and the strongest fluctuation appears at the center part of each incoherent domain. Accordingly, Fig 1(f) shows that the variance *σ*_*k*_ stays at its highest value at the center part of the incoherent domains. Furthermore, Fig 1(f) shows *σ*_*k*_ = 0 in the two large coherent domains while nonzero *σ*_*k*_ in the M- and S-domains. Fig 1(g) shows a typical spatiotemporal plot of the variable *X*_*k*_ for the 2-frequency chimera state. To be mentioned, synchronous state is stable at the parameters in Fig 1. That is, the 2-frequency chimera state coexists with the synchronous state. Moreover, the attraction basin of the synchronous state is overwhelmingly larger than that of the 2-frequency chimera state. Consequently, Eq (1) always builds up the synchronous state for arbitrary initial conditions and the establishment of the 2-frequency chimera state requires deliberately prepared initial conditions. However, at certain range of *θ* such as *θ* ∈ [0.6, 1.1], the synchronous state might be unstable in Eq (1) and chimera states can be easily built up for random initial conditions (The results on that are beyond the scope of this work and are not presented here.). Using these chimera states as initial conditions, we find that the 2-frequency chimera states are possible to be realized. For example, the 2-frequency chimera state in Fig 1 is generated using the chimera state at *σ* = 0.09 and *θ* = 0.6.

**Fig 2:**
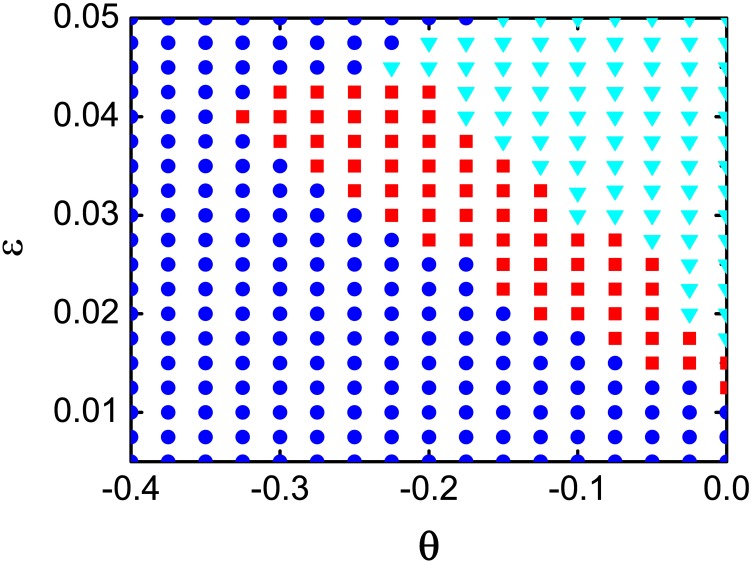
Stability diagram. The stability diagram of the 2-frequency chimera state in the *θ* − *ϵ* plane. The 2-frequency chimera state, the 2-cluster chimera state, and the synchronous state are distinguished by the red squares, the cyan triangles, and blue circles, respectively. Other parameters are same as in Fig 1.

To gain an overall view of 2-frequency chimera states, we explore the *θ* − *ϵ* plane in the range [−0.4, 0] × [0.005, 0.05]. We use the chimera state in Fig 1 as initial conditions and integrate Eq (1) for 10^5^ time units. After this interval, if the final state possesses a profile of mean phase velocity with two different coherent frequencies, we classify the 2-frequency chimera state as stable. The stability diagram of the 2-frequency chimera states is presented in Fig 2. We observe a narrow stripe extending from *θ* ≃ −0.325 and *ϵ* ≃ 0.04 down to *θ* ≃ 0 and *ϵ* ≃ 0.0125. Ordinary chimera state with two coherent clusters are developed on the right side of the stable regime of the 2-frequency chimera state. In contrast, the coherent states including synchronous state and travelling wave states appear on the left side of the stability regime (To be noted, the synchronous state is always stable in Fig 2 if arbitrary initial conditions are adopted.)

**Fig 3:**
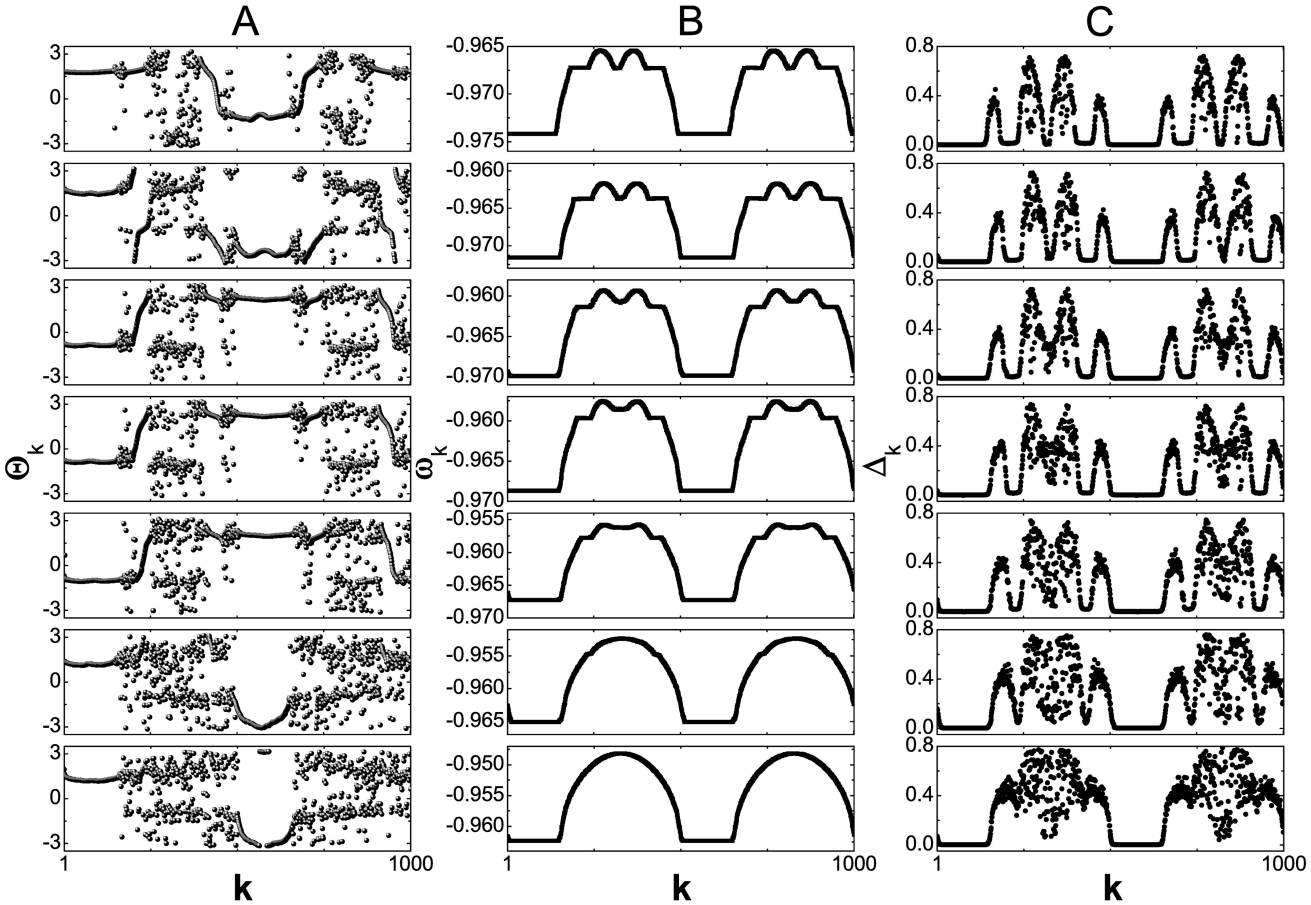
Bifurcation scenario. Typical bifurcation scenario for the 2-frequency chimera state with *θ* = −0.1. For each value of the coupling strength *ϵ* (increasing from the top to the bottom, *ϵ* = 0.02, 0.0225, 0.024, 0.025, 0.026, 0.0275, and 0.03, respectively) the snapshots of Θ_*k*_ (column A), the profile of the mean phase velocity *ω*_*k*_ (column B), and the profile of Δ_*k*_ (column C) are shown. Other parameters are same as in Fig 1.

Now we investigate the transition between the 2-frequency chimera state and the 2-cluster chimera state. The typical bifurcation scenario of the transition between them is presented in Fig 3, where we fix *θ* = −0.1 and increase the coupling strength *ϵ* from 0.02 to 0.03. Each row in Fig 3 has been made for different coupling strengths, starting with the chimera state in Fig 1 as initial conditions. The column *A* presenting the snapshots of the phases of oscillators after transient time shows that increasing *ϵ* turns a 2-frequency chimera state to a 2-cluster one. During the process, the two large coherent domains remain while the other small coherent domains are eliminated. Oscillators in different coherent domains are in anti-phase for the 2-cluster chimera state, which provides an explanation for the anti-phase between two large coherent domains in a 2-frequency chimera state. The columns *B* and *C*, presenting the profiles of the mean phase velocity *ω*_*k*_ and the profiles of Δ_*k*_, respectively, suggest that the transition is a continuous one. With the coupling strength *ϵ* increase, the sizes of the small coherent domains vanish gradually and, interestingly, the small coherent domains in the 2-frequency chimera state locate in the center part of the incoherent domains in the 2-cluster chimera state. The phenomenon that new coherent domains emerge out of incoherent domain with parameter change has been observed in Ref. [11]. Different from the 2-frequency chimera state, there the new coherent domains share the same mean phase velocity with previous ones. The continuous transition between the 2-frequency chimera state and the 2-cluster chimera state can be supported by using the 2-cluster chimera states at *ϵ* = 0.03 as initial conditions. With the coupling strength *ϵ* decrease, Fig 3 can be reproduced.

## Conclusion

In conclusion, we have investigated nonlocally coupled Brusselators in a ring. We reported a new type of chimera states, 2-frequency chimera state. In a 2-frequency chimera state, there exist two types of coherent domains and oscillators in different types of coherent domains have different mean phase velocities. We explored the stability diagram of the 2-frequency chimera state in the parameter *θ* − *ϵ* plane. We studied the transition between the 2-frequency chimera state and 2-cluster chimera state and found that the transition is a continuous one. The discovery of the 2-frequency chimera state may shed light on the future studies on chimera states.
